# Impact of a training program incorporating cardiac magnetic resonance imaging on the accuracy and reproducibility of two-dimensional echocardiographic measurements of left ventricular volumes and ejection fraction

**DOI:** 10.1186/s12947-019-0173-z

**Published:** 2019-10-31

**Authors:** Yosuke Nabeshima, Hidehiro Namisaki, Toshihiro Teshima, Yasuhiko Kurashige, Akiko Kakio, Azusa Fukumitsu, Yutaka Otsuji, Masaaki Takeuchi

**Affiliations:** 1 Second Department of Internal Medicine, University of Occupational and Environmental Health, School of Medicine, 1-1 Iseigaoka, Yahatanishi-ku, Kitakyushu, 807-8556 Japan; 2 Department of Laboratory and Transfusion Medicine, Hospital of University of Occupational and Environmental Health, School of Medicine, Kitakyushu, Japan; 3 Department of Laboratory, Saiseikai Fukuoka General Hospital, Fukuoka, Japan; 4 Department of Laboratory, Shin Koga Hospital, Kurume, Japan; 5 Department of Laboratory, Kyushu Rousai Hospital, Kitakyushu, Japan; 6 Department of Laboratory, JCHO Kyushu Hospital, Kitakyushu, Japan

**Keywords:** Echocardiography, Quality improvement, Cardiac magnetic resonance, Coverage probability

## Abstract

**Background:**

Left ventricular (LV) ejection fraction (LVEF) assessed by two-dimensional echocardiography (2DE) is the most widely used parameter for clinical decision-making, but reproducibility and accuracy problems remain. We evaluated the usefulness of a novel training program based on cardiac magnetic resonance (CMR) imaging to obtain more reliable values of 2DE-derived LVEF and LV volumes.

**Methods:**

Fifty-four sonographers from five hospitals independently measured LV volumes and LVEF using the same 2DE images from 15 patients who underwent CMR and 2DE. After receiving a lecture from an expert on how to properly trace the LV endocardium, each sonographer voluntary performed the measurements using the same datasets, and was invited to perform the same analysis for additional patients. The effect of the training intervention was evaluated using the coefficient of variation (CV) and coverage probability (CP).

**Results:**

Before the intervention, the LV volumes were significantly underestimated and the LVEF was significantly overestimated compared to the CMR results; however, these differences were reduced after the intervention. In particular, the CP (0.52 vs. 0.76, *p* < 0.001) for the LVEF showed significant improvement. However, the degree of improvement differed among institutions, and the CV actually became worse in two hospitals after the intervention. Level of experience and self-practice was associated with the reproducibility after the intervention.

**Conclusions:**

A training program using CMR as a reference improved the accuracy of 2DE-determined LV measurements. Since the degree of improvements differed among hospitals, individualization of training programs and periodical objective evaluation may be required to reduce inter-institutional variability.

## Background

Echocardiography plays a pivotal role in the diagnosis and management of cardiovascular diseases and other pathologies [1, 2]. In particular, assessing the left ventricular (LV) ejection fraction (LVEF) is one of the most common reasons to perform echocardiography. Because the LVEF is an important parameter to guide appropriate medication and device therapy [3, 4], the accuracy and reproducibility of LVEF measurement is of paramount importance for clinical decision-making [5, 6]. However, the reproducibility of LV volume measurements remains a serious concern due to the fact that manual tracing of the LV endocardial border using two-dimensional echocardiography (2DE) produces non-negligible measurement variability [7]. Thus, the American Society of Echocardiography recommends the annual assessment of observer variability of LVEF [8]. However, the lack of a reference standard makes this approach ineffective for reducing the inter-institutional variability and improving accuracy. The potential solutions to resolve this problem are to (1) use fully automated LV quantification software or (2) establish a training program to enhance the accuracy and reproducibility of the measurements. Although the former approach is more robust and straightforward, it requires specific equipment that cannot be used in routine echocardiographic laboratories.

Cardiac magnetic resonance (CMR) imaging is a reference standard to measure both LV volumes and LVEF [9]. If both CMR and 2DE are conducted on the same day, the LV volumes and LVEF assessed by CMR can serve as references for the corresponding 2DE measurements. Thus, we hypothesized that a training program aimed at obtaining 2DE LV volumes and LVEF values similar to CMR measurements would help to improve inter-observer and inter-institutional variability and consequently the overall accuracy of the assessment.

Accordingly, we aimed to (1) clarify the inter-observer and inter-institutional variability of 2DE-determined LV volumes and LVEF in a large number of examiners and to assess their accuracy against CMR measurements; (2) evaluate whether intervention via a training program including expert guidance would improve reproducibility and accuracy; and (3) determine sonographer’s characteristics on reproducibility after the intervention.

## Methods

This study was approved by the Institutional Review Board of the University of Occupational and Environmental Health, School of Medicine. All examiners provided informed consent for participation in this study.

### Patients

We selected a consecutive series of 15 patients who underwent clinically indicated CMR examinations and also agreed to undergo 2DE examinations on the same day among the CMR database of our laboratory. We did not exclude any patients who had poor 2DE image quality. We then aimed to select an additional 15 patients with propensity score matching of LV end-diastolic volume (LVEDV) and LVEF on the CMR and 2DE image quality from the same CMR database. However, one of the patients among the first 15 cases did not have an appropriate propensity score match in the database. Thus, we did not use this patient for the comparative analysis before and after the intervention.

### Acquisition and analysis of 2DE images

All 2DE images were acquired by one expert sonographer using a commercially available ultrasound machine and equipment (iE33, Philips Medical Systems, Andover, MA, USA). Echocardiographic image acquisition was systematically performed according to the American Society of Echocardiography guidelines [8] with breath-holding in all patients, from which we used apical LV-focused 4- and 2-chamber views for the analysis. LV volumes (LVEDV, LV end-systolic volume: LVESV) and LVEF were measured by tracing the LV endocardial border at end-diastole and at end-systole on the apical 4- and 2-chamber views and were calculated using the modified biplane Simpson method (ImageArena, TomTec Imaging Systems, Unterschleissheim, Germany). Image quality was evaluated according to the visualization of the LV endocardial border in LV 18-segment model (good: 0–2 segments were not visible, fair: 3–5 segments were not visible, and poor: > 5 segments were poorly visible).

### Acquisition and analysis of CMR images

CMR imaging was performed using a 3-T scanner (Discovery 750 W; GE Healthcare, Milwaukee, WI) with a phased-array cardiovascular coil. Retrospective electrocardiography-gated localizing spin-echo sequences were used to identify the long axis of the heart. Steady-state free precision (SSFP) dynamic gradient-echo cine loops were acquired by retrospective electrocardiographic gating and parallel imaging techniques during 10- to 15-s breath-holds with the following general parameters: 8-mm slice thickness of the imaging planes, 40 × 40-cm field of view, 200 × 160-scan matrix, 50° flip angle, 3.8/1.7-ms repetition/echo times, and 20 reconstructed cardiac phases. Eight to 16 short-axis slices from the base of the heart to the apex, and three standard long-axis views were recorded in each patient.

CMR LV volumes and LVEF were measured by the same operator (the expert that visited each hospital for training intervention) via feature tracking analysis (2D CPA MR; TomTec Imaging Systems, Unterschleissheim, Germany). In three apical long-axis SSFP images, the LV endocardial border was manually traced at the end-diastolic frame. Then, the feature tracking software propagates the endocardial contour and follows its tissue features throughout a cardiac cycle to generate LV volume curves from which LVEDV, LVESV, and LVEF were determined automatically.

### Examiners

We invited cardiac sonographers working in five different hospitals to participate in the study. We defined an expert sonographer as one with > 10 years of experience in echocardiography and a novice sonographer one having ≤10 years’ experience [10]. Moreover, we defined an active sonographer as the sonographer who performs > 1000 echocardiography examinations/year.

### Protocol 1

Anonymized 2DE apical 4- and 2-chamber DICOM images for 15 patients were sent to each participating hospital. There were only two DICOM images for each patient, with three consecutive cardiac cycles for each image. Each sonographer selected one cardiac cycle from the three beats and determined both the end-diastolic and end-systolic frames. Subsequently, the sonographer traced the LV endocardial border, which was used for calculation of LVEDV, LVESV, and LVEF. After finishing the measurements, all measurement data were sent to the core laboratory. Differences in LV volumes and LVEF between 2DE and CMR examinations were determined for each participant. We also analyzed the inter-reader and inter-institutional variability.

### Protocol 2

**Fig. 1:**
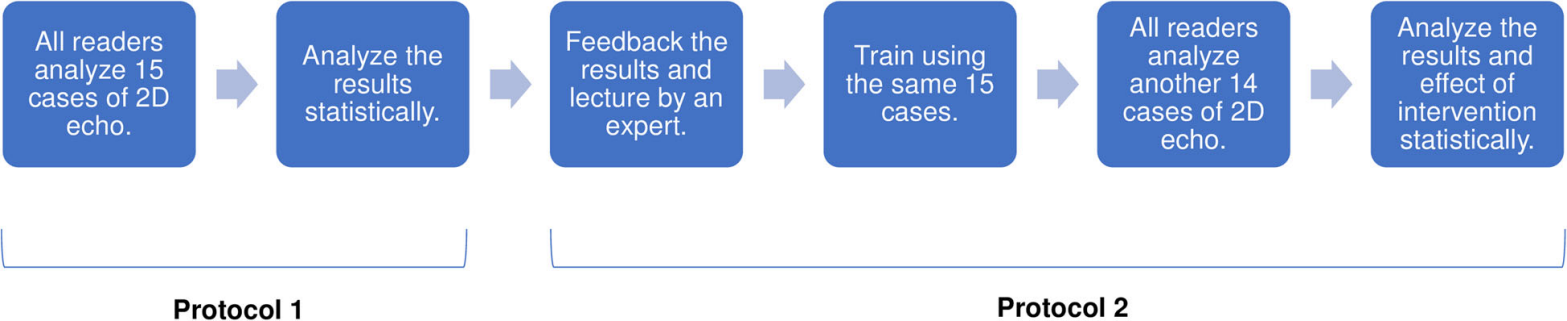
Process of quality improvement. Quality improvement process to assess inter-reader and inter-institutional variability and achieve improvement of reproducibility and accuracy

The LVEDV, LVESV, and LVEF values for all 15 patients determined by CMR were given as feedback to each sonographer. An expert (MT) then visited each hospital and gave a 4-h hands-on lecture on how to best trace the LV endocardium border to obtain similar values to the CMR measurements. The lecture was also video recorded and uploaded to a webpage where each participant was able to access. We allocated 3 months for the sonographers to individually or jointly practice LV border tracing using the same datasets of protocol 1. This practice period was completely arbitrary and individually based. When the 3-month practice period was finished, we sent the propensity score matched 2DE apical 4- and 2-chamber DICOM images from the additional 14 patients to the same sonographers who were invited to repeat the analysis. The same statistical analysis was performed in the core laboratory on the data as conducted for protocol 1 to assess the impact of the intervention. The study protocol is schematically summarized in Fig. 1.

### Statistical analysis

Continuous data are expressed as the mean ± standard deviation (SD). Categorical data are presented as a number or percentage. The t-test or Wilcoxon rank sum test was used to evaluate the differences in continuous variables between two groups. Friedman’s analysis with post-hoc comparison was used to determine differences in the 2DE assessment between each hospital and from the CMR results. The coefficient of variation (CV) was used to evaluate reproducibility [11], defined as the SD divided by the mean value. Coverage probability (CP) was used to evaluate the accuracy of echocardiographic measurements compared to CMR results. CP represents the probability of the absolute differences falling within the acceptable difference. Although it is generally used for assessing reproducibility [12, 13], we considered CP to be a useful parameter to guide review and retraining efforts. In accordance with previous studies, the acceptable difference was defined as 30 mL for LVEDV and LVESV and 10% for LVEF [12, 13]. A two-sided *p* value < 0.05 was defined as statistically significant. All statistical analyses were performed using commercial software (JMP, version 14; SAS Institute, Inc., Cary, NC, USA) and R version 3.1.0. (The R Foundation for Statistical Computing, Vienna, Austria) with the party package.

## Results

A total of 54 sonographers agreed to participate the study. Table 1 shows characteristics of sonographers in each hospital. All five hospitals play an important role for the management of acute cardiovascular care. A core laboratory was located in Site A. Except for Site B, other four hospitals have a cardiovascular center and department of cardiovascular surgery. The median sonographers’ experience with echocardiography was 11 years. Table 2 depicts the clinical characteristics of the study population included in protocols 1 and 2. Among 29 echocardiography examinations, image quality analysis revealed good in 5 cases (17%), fair in 11 cases (38%), and poor in 13 cases (45%), respectively. Among the 54 sonographers participating in protocol 1, three sonographers did not participate in protocol 2 due to pregnancy (*n* = 1) and moving to a different hospital (*n* = 2). Thus, we used the results from 51 sonographers.

**Table 1:** Sonographers and hospitals’ characteristics

	Type of hospital	Number of beds	Number of studies by year	Number of sonographers	Number of participants	Experience (years)	Expert	Active sonographer	Number of participants doing self-practice
Site A	University hospital	700	8800	10	9	8 (4, 15)	33%	56%	67%
Site B	Public hospital	450	5400	7	7	13 (6, 26)	67%	0%	100%
Site C	Private hospital	459	12,000	29	22	12 (8, 15)	67%	27%	60%
Site D	Private hospital	380	10,000	8	6	10 (7, 17)	40%	100%	0%
Site E	Public hospital	575	6500	10	10	9 (5, 16)	40%	70%	0%

**Table 2:** Patients’ characteristics

	Protocol 1 (*n* = 15)	Protocol 2 (*n* = 14)
Clinical Diagnosis		
Valvular Heart Disease	4	2
Ischemic Heart Disease	2	5
Dilated Cardiomyopathy	2	4
Hypertrophic Cardiomyopathy	0	1
Secondary Cardiomyopathy	4	0
Arrhythmia	2	1
Others	1	1
Image Quality		
Good	2	3
Fair	6	5
Poor	7	6
CMR measurements		
EDV (mL)	187 ± 95	176 ± 70
ESV (mL)	116 ± 77	113 ± 62
EF (%)	41 ± 15	40 ± 15

*EDV* End-diastolic volume, *ESV* End-systolic volume, *EF* Ejection fraction, *CMR* Cardiac magnetic resonance

### Protocol 1

LV volume and LVEF measurements of each hospital and the CMR examination are summarized in Table 3. LV volumes measured by 2DE were significantly underestimated and LVEF was significantly overestimated compared to the corresponding values of CMR measurements in each hospital and for the whole cohort. However, the degree of this discordance differed substantially among hospitals (Supplemental Table 1). The percentage of underestimation of LVESV was higher than that of LVEDV, resulting in the significant overestimation of LVEF in all five hospitals. Although the CV of LVEDV, LVESV, and LVEF in each hospital ranged from 11 to 16%, from 13 to 21%, and from 8 to 13%, respectively, the corresponding CVs in the whole cohort were 18, 25, and 13%, respectively, suggesting the presence of large inter-institutional variability. The CP of LVEDV ranged from 0.21 to 0.56 in each hospital, indicating that only one-quarter to one-half of the measurements fall into the acceptable range of LVEDV differences (≤30 mL). Similarly, the CP for LVEF ranged from 0.40 to 0.77. These results reflected the different habits of tracing the endocardial border at end-diastole and end-systole among individual hospitals, pointing to the need for the standardization.

**Table 3:** Echocardiographic measurements and statistical results

	Site A	Site B	Site C	Site D	Site E	All	CMR
Protocol 1 (*n* = 15)							
Measurements							
EDV (mL)	164 ± 72 †	171 ± 85 †	161 ± 76 †	128 ± 65 †	130 ± 66 †	153 ± 75 †	187 ± 95
ESV (mL)	98 ± 57 †	100 ± 73 †	83 ± 55 †	62 ± 40 †	69 ± 46 †	83 ± 56 †	116 ± 77
EF (%)	44 ± 16 †	47 ± 18 †	52 ± 16 †	55 ± 15 †	51 ± 16 †	50 ± 16 †	41 ± 15
CV (%)							
EDV	11.5 ± 3.8	15.9 ± 6.9	13.8 ± 3.6	11.3 ± 3.8	10.5 ± 3.2	17.8 ± 3.6	–
ESV	16.1 ± 7.3	21.0 ± 8.1	17.3 ± 5.2	12.9 ± 6.4	14.0 ± 5.9	24.5 ± 6.2	–
EF	10.9 ± 5.4	13.4 ± 8.8	9.4 ± 4.4	8.9 ± 4.3	8.3 ± 3.4	13.1 ± 5.5	–
CP							
EDV	0.56	0.54	0.51	0.21	0.22	0.44	–
ESV	0.73	0.75	0.57	0.36	0.42	0.57	–
EF	0.77	0.67	0.43	0.40	0.57	0.54	–
Protocol 2 (*n* = 14)							
Measurements							
EDV (mL)	160 ± 53 †	166 ± 50	187 ± 58 †	149 ± 46 †	157 ± 53 †	170 ± 56 †	176 ± 70
ESV (mL)	99 ± 50 †	106 ± 50 †	116 ± 52	84 ± 42 †	97 ± 49 †	105 ± 51 †	113 ± 62
EF (%)	41 ± 16	39 ± 16	40 ± 15	46 ± 16 †	41 ± 16	41 ± 16 †	40 ± 15
CV (%)							
EDV	9.2 ± 2.8	11.2 ± 5.0	11.6 ± 3.1	9.8 ± 2.4	16.7 ± 4.8	15.1 ± 3.5	–
ESV	11.9 ± 5.0	14.5 ± 5.3	17.0 ± 7.0	12.7 ± 4.8	23.0 ± 7.2	20.8 ± 6.4	–
EF	10.9 ± 5.8	14.9 ± 9.2	15.5 ± 4.6	9.3 ± 6.3	14.6 ± 5.3	15.3 ± 4.9	–
CP							
EDV	0.59	0.55	0.49	0.50	0.54	0.53	–
ESV	0.75	0.70	0.68	0.61	0.66	0.68	–
EF	0.83	0.76	0.74	0.71	0.78	0.76	–

†: *p* value < 0.05 against CMR

*EDV* End-diastolic volume, *ESV* End-systolic volume, *EF* Ejection fraction, *CMR* Cardiac magnetic resonance

### Protocol 2

**Fig. 2:**
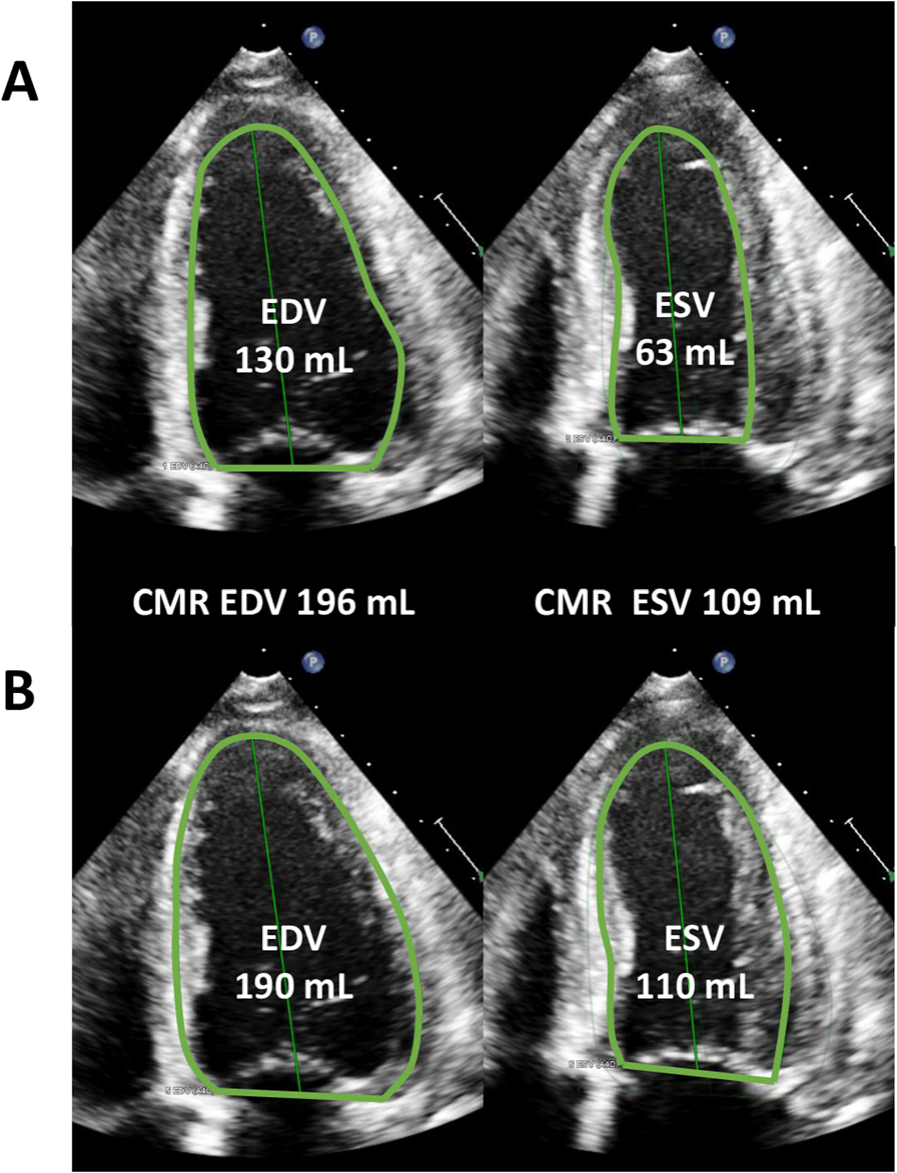
Typical pattern of tracing the endocardium before (**a**) and after (**b**) the lecture. Participants were lectured to trace the endocardium more outwardly. Specifically, the importance of tracing the borderline of the compacted and non-compacted layer was emphasized

Figure 2 depicts a representative case showing LV endocardial border tracing before and after the practice. LV volume and LVEF measurements of each hospital and CMR examination after the intervention are summarized in Table 3. Although most hospitals still showed underestimation of LVEDV and LVESV, the degree of underestimation became smaller, and the difference was no longer statistically significant in some hospitals. All but one hospital reported LVEF values similar to those measured with CMR. Although the CP values of LVEDV were still low (0.49–0.59), those of LVEF ranged from 0.71 to 0.83; in particular, site A provided a CP value > 0.80.

Table 4 indicates the comparison of several reproducibility parameters before and after the intervention. The CP significantly improved after the intervention in most hospitals; however, the CV actually became worse in two hospitals. In particular, at sites C and E, despite significant improvements in the CP of LV volumes and LVEF, the CV of LVEF became significantly worse from 9.9 to 15.5% and from 8.7 to 14.6%, respectively.

**Table 4:** Change of statistical parameters before and after intervention

	Site A (*n* = 9)			Site B (*n* = 6)			Site C (*n* = 21)			Site D (*n* = 5)			Site E (*n* = 10)			All (*n* = 51)		
	P1	P2	p	P1	P2	p	P1	P2	p	P1	P2	p	P1	P2	p	P1	P2	p
CV (%)																		
EDV	11.8	9.2	0.072	14.6	11.2	0.157	14.0	11.6	0.113	11.6	9.8	0.165	10.8	16.7	< 0.001	18.0	15.1	0.279
ESV	16.2	11.9	0.107	19.9	14.5	0.070	17.6	17.0	0.784	13.0	12.7	0.919	14.3	23.0	0.002	24.7	20.8	0.761
EF	11.0	10.9	0.957	13.9	14.9	0.786	9.9	15.5	0.005	9.4	9.3	0.987	8.7	14.6	0.008	13.1	15.3	0.022
CP																		
EDV	0.57	0.59	0.760	0.57	0.55	0.859	0.53	0.49	0.384	0.23	0.50	0.038	0.24	0.54	< 0.001	0.46	0.53	0.043
ESV	0.73	0.75	0.500	0.75	0.70	0.655	0.56	0.68	0.002	0.37	0.61	0.011	0.42	0.66	< 0.001	0.57	0.68	< 0.001
EF	0.78	0.83	0.211	0.65	0.76	0.151	0.39	0.74	< 0.001	0.36	0.71	0.010	0.54	0.78	< 0.001	0.52	0.76	< 0.001

*P1* Protocol 1 (number of patients was 14), *P2* Protocol 2 (number of patients was 14), *EDV* End-diastolic volume, *ESV* End-systolic volume, *EF* Ejection fraction, n, number of sonographers participating in both protocols

**Fig. 3:**
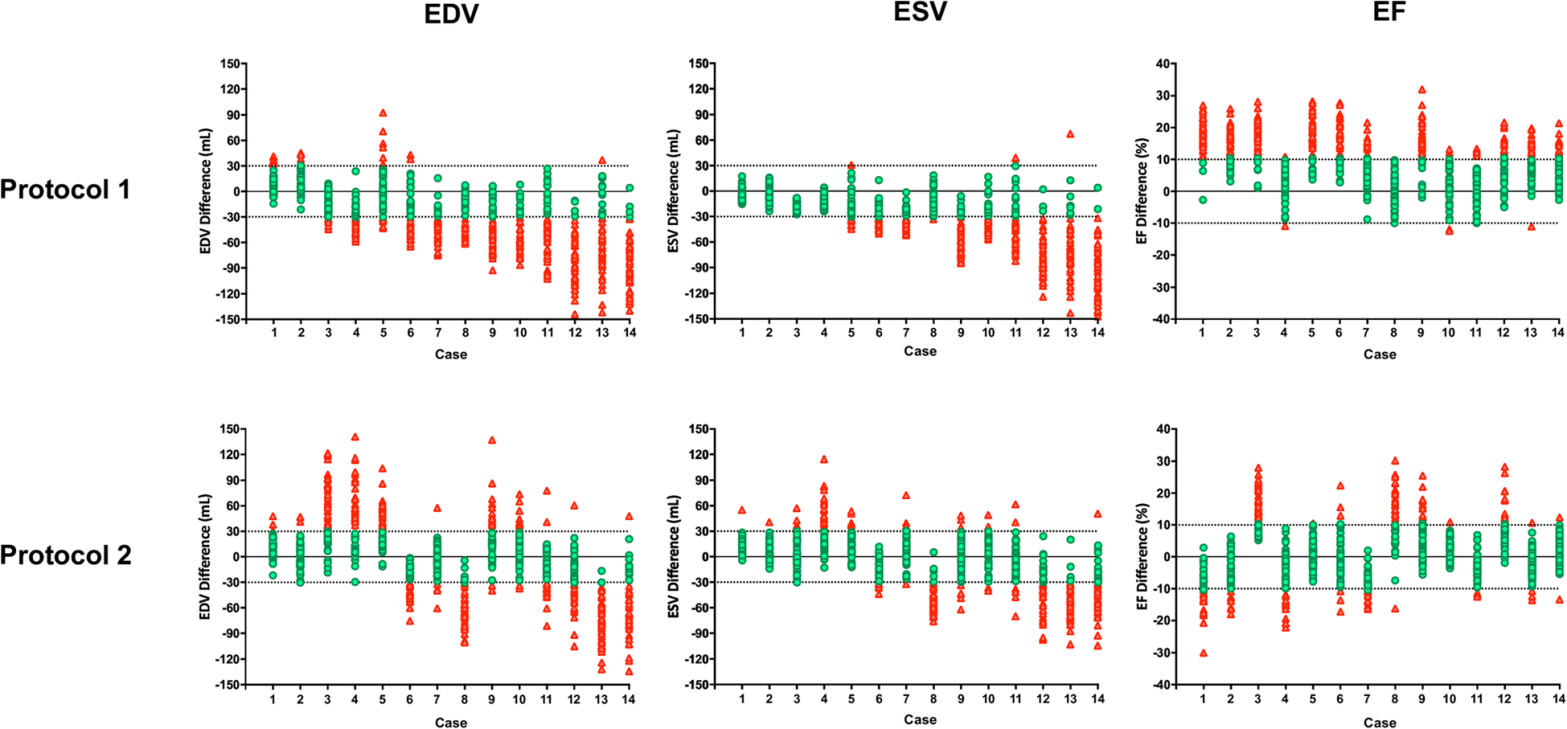
Measurement of differences between 2D echocardiography and cardiac magnetic resonance in each case. Cases are sorted according to the degree of LVEDV determined by CMR; case 1 has the smallest LVEDV and case 14 has the largest LVEDV. Green plots indicate differences falling within the acceptable difference, and red plots indicate differences outside the acceptable difference. CMR, cardiac magnetic resonance; LVEDV, left ventricular end-diastolic volume

**Fig. 4:**
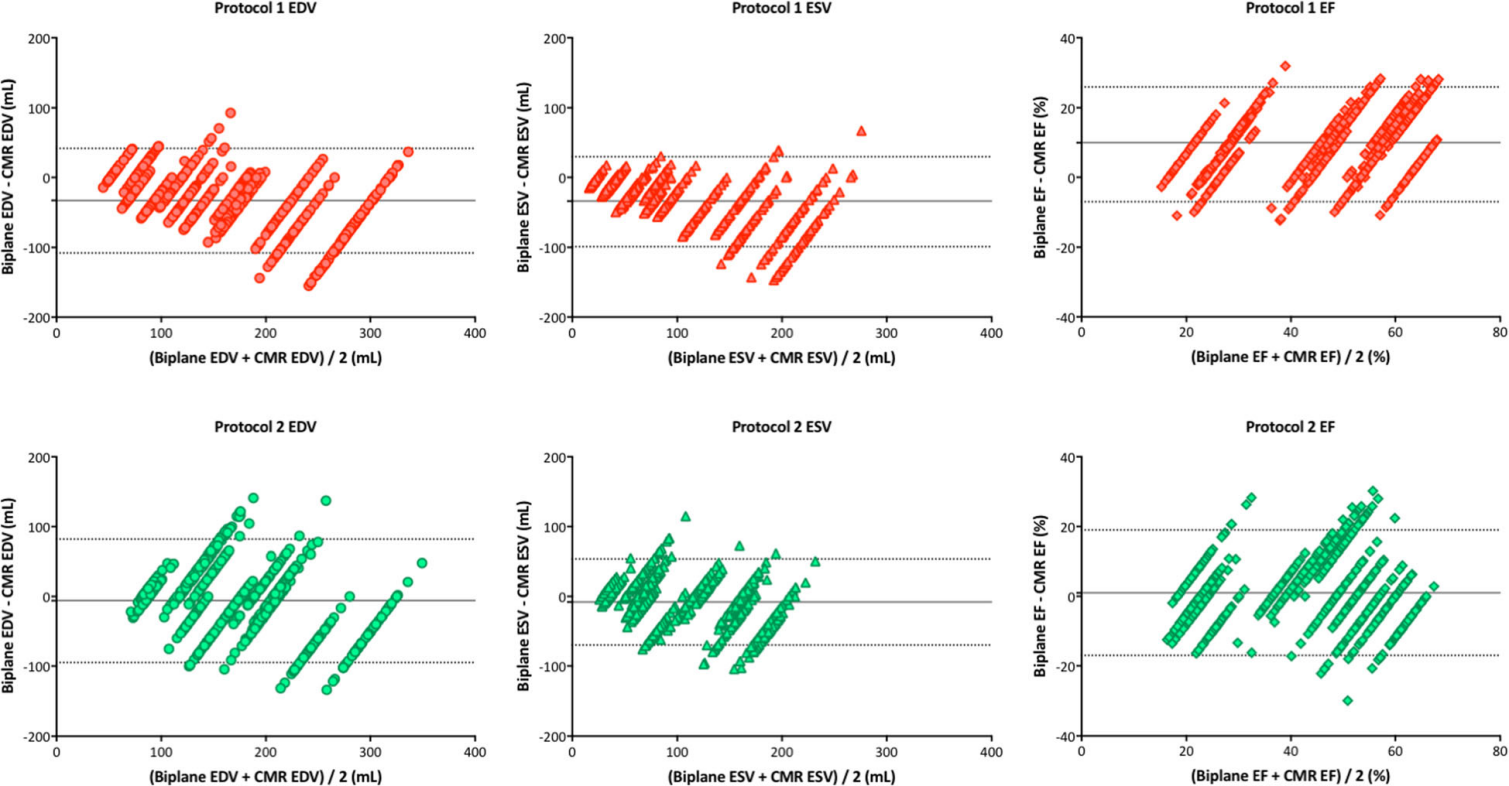
Bland-Altman plots of measurements comparison between 2D echocardiography and cardiac magnetic resonance in each case. Solid lines indicate mean difference and broken lines indicate upper and lower 95% limit of agreement

Figure 3 shows the measurement biases of LVEDV, LVESV, and LVEF between the two methods (2DE – CMR) for each case sorted according to the values of CMR determined LV volumes, with the LV volumes gradually increasing from the left-sided case to the right-sided case. The green plots show measurement differences falling within the acceptable range and the red plots show differences outside the acceptable range: the number of green plots divided by the number of all plots indicates the CP value. The plots of protocol 1 indicate that larger LV volumes were associated with greater underestimation of 2DE determined both LVEDV and LVESV. Bland-Altman analysis also showed that the degree of underestimation of LV volumes especially in large left ventricles became smaller after the intervention (Fig. 4). Since absolute differences between the two methods of measuring LVEDV and LVESV appeared to be similar, there was consistent overestimation of LVEF before the intervention. However, the underestimation of LV volumes became smaller after the intervention, especially for LVESV. In addition, the 2DE measurements were actually overestimated for both LVEDV and LVESV in cases with a small left ventricle. These changes resulted in the LVEF from 2DE becoming similar to CMR measurements.

**Fig. 5:**
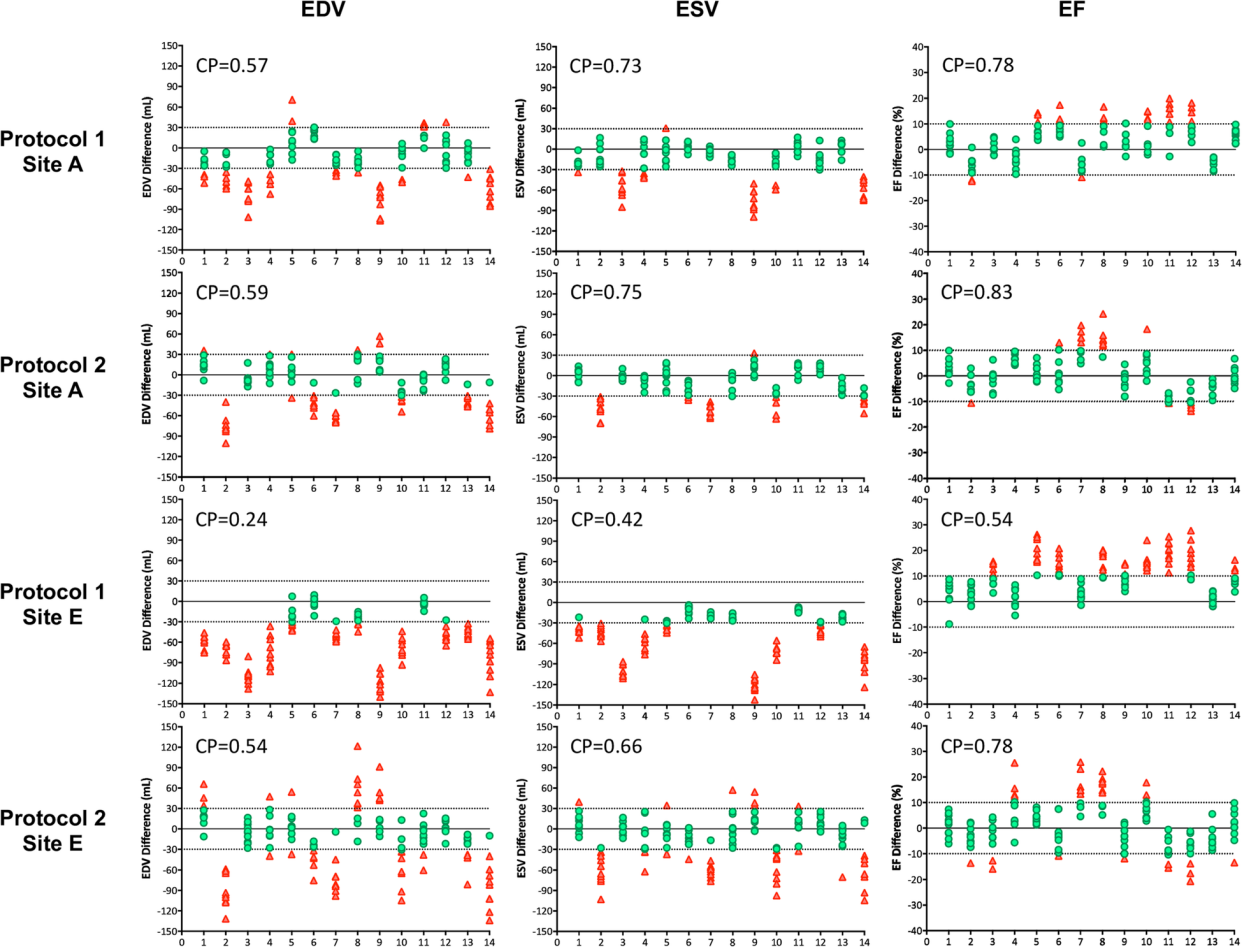
Difference of the effect of the training program between sites A and E

CP analysis revealed that the effect of the intervention was different among the hospitals. Although the tracing habit of the sonographers working at sites C, D, and E obviously changed following the intervention, the effect was much smaller at sites A and B. Figure 5 provides a representative example of the difference of the effect of the intervention between sites A and E. The effect of intervention was more obvious at site E, especially for the measurements of LVESV and LVEF.

### Sonographer characteristics and reproducibility

Years of experience for echocardiography was not different among five hospitals (*p* = 0.801). The prevalence of the expert sonographer was not different (*p* = 0.409) (Table 1). Although total number of echocardiography examinations performed in each institution was the highest in Site C, mean number of echocardiography examinations per one sonographer was the lowest in Site C (*n* = 414) and followed by Site E (*n* = 650).

All but four sonographers attended the hands-on lecture or watched the web video lecture. However, less than half of sonographers (47%) actually performed the self-practice for LV endocardial border tracing before the protocol 2.

Table 5 indicates the effect of intervention according to the characteristics of sonographers. Although CP values before and after the intervention were not different between the expert group and the novice group, the expert group had significantly lower CV values of LVEDV and LVESV than those of the novice group after the intervention. Active sonographers had a larger improvement CP values of EDV between before and after the intervention compared with inactive sonographers. Sonographers who performed self-practice had a significantly higher CP values of LVEDV and LVESV compared with sonographers who did not perform self-practice before the intervention. Sonographers who did not perform self-practice showed significantly higher values of CV for LVEDV and LVESV after the intervention than those who performed self-practice.

**Table 5:** Effect of training to specific groups

	Expert	Novice	p	Active	Inactive	p	Practiced	Not practiced	p
CP									
Protocol 1									
EDV	0.45	0.45	0.892	0.37	0.52	0.013*	0.54	0.37	0.005*
ESV	0.59	0.56	0.588	0.51	0.63	0.030*	0.66	0.50	0.002*
EF	0.51	0.57	0.337	0.53	0.55	0.771	0.58	0.50	0.174
Protocol 2									
EDV	0.54	0.53	0.644	0.54	0.53	0.640	0.54	0.54	0.956
ESV	0.69	0.69	0.898	0.67	0.70	0.446	0.71	0.67	0.264
EF	0.77	0.77	0.978	0.74	0.79	0.176	0.78	0.75	0.334
ΔEDV	0.09	0.08	0.939	0.18	0.01	0.019*	0.00	0.17	0.018*
ΔESV	0.10	0.13	0.667	0.16	0.07	0.123	0.05	0.17	0.030*
ΔEF	0.25	0.20	0.334	0.21	0.24	0.686	0.20	0.25	0.401
CV (%)									
Protocol 1									
EDV	17.1	18.7	0.084	19.5	15.7	< 0.001*	13.3	19.7	< 0.001*
ESV	23.6	26.0	0.135	28.2	21.5	< 0.001*	15.4	14.9	0.460
EF	13.9	13.1	0.492	12.9	13.9	0.221	14.7	11.7	0.010*
Protocol 2									
EDV	14.1	15.9	0.025*	13.5	14.1	0.431	13.6	16.1	0.003*
ESV	18.8	22.2	0.002*	19.5	19.3	0.806	18.7	21.9	0.009*
EF	15.5	15.2	0.658	14.8	15.4	0.462	15.4	14.9	0.460

"*" means *p* < 0.05

## Discussion

The main strengths of our study include the relatively large number of participants, new insights concerning CP usage, and provision of a novel training program incorporating CMR as a reference.

Inter-observer variability is a major limitation of echocardiography, especially for assessing LV volumes and LVEF [7, 12, 14, 15]. Accurate determination of the LV endocardial border is critical for the reliable calculation of these parameters; however, there is no clear consensus regarding the precise tracing border, which is one of the most important causes of inter-observer variability. One potential solution is to use corresponding values from other imaging modalities as a reference. Owing to its high spatial resolution and tissue contrast, CMR SSFP images provide clearer visualization of the LV endocardial border, and the LV volumes and LVEF measured by CMR are associated with reduced inter-observer variability [16]. Several previous studies indicated that 2DE systematically underestimates LV volumes and overestimates LVEF compared to CMR due to foreshortening of 2D cut-planes and individual habits of tracing the more inner part of the LV myocardium [17]. The latter cause is more important, because even a 1-mm difference in tracing the LV endocardial border could produce a significant change in the obtained LV volume [18]. Since CMR provides reference values of LVEDV and LVESV for each patient [19], this offers an opportunity to examine precisely where to trace so as to obtain similar values for LV volumes on 2DE images. In the present study, we evaluated the usefulness of this training program for obtaining more reliable measurements of LV volumes and LVEF using 2DE. As expected, we found significant inter-observer and inter-institutional variability in LV volume measurements before the intervention. Overall, our training program had a favorable impact with a diverse range of echocardiographic image quality. However, the degree of this effect was different among institutions, and thus the approach was not sufficient to overcome inter-institutional variability in LVEF measurements.

When the LV endocardial border is traced using the biplane Simpson method, the eyeball EF (visual EF) must also be taken into consideration [20]. LVEF determination by visual EF might differ for each sonographer as well as in each institution [21]. Indeed, although the mean values of LVEF differed among each institution, the CV of each institution was distributed in a relatively narrow range (8.3–11.0%), except for one hospital (14.1%), before the intervention. These results are consistent with those of a recent study conducted by Khouri et al. [13], indicating that there are site-specific criteria for visual EF determination. Thus, without improvement of this inter-institutional visual EF variability, inter-institutional LVEF variability using Simpson’s method may not be reduced. Although an expert lectured on how to effectively trace the LV endocardial border and discussed the visual EF in selected cases, there was significant deterioration of the CV for LVEF after the intervention in some institutions. One possible reason for this effect is that the training time was too short for every sonographer to change their established habits concerning tracing of the LV endocardium with confidence in every case. This may also indicate that even if the intervention led to improvement of measurement accuracy in comparison to CMR, it might also have caused confusion for some sonographers, resulting in an overall loss of consistency in some institutions (sites C and E). A long-standing training program, increasing sonographers’ daily practice and repeated educational hands-on lectures may be required to overcome this problem.

We also analyzed the relationship between sonographers’ characteristics and reproducibility. Expert sonographers (> 10 years’ experience) showed better CVs of LVEDV and LVESV after the intervention than novice sonographers. CVs of LVEDV and LVESV in sonographers who perform more than 1000 examinations per year markedly improved after the training. Importantly, sonographers who performed the self-practice showed significantly lower CV values before and after the intervention compared with the sonographers who did not perform the self-training. Our results suggest that not only clinical experience but also voluntary self-practice are important to improve tracing habitus with keeping the range of CV values among the groups. Medvedofsky et al. [22] has already reported that experience is important for reproducibility of LVEF measurements in which study one expert echocardiographer and three novices who were not intervened participated. Our results were consistent with this study and novelty of our study is that experience of sonographer is also associated with the reproducibility.

This study further demonstrates the potential utility of CP as a quality control parameter. CP has been used as an indicator of inter-observer variability of echocardiographic measurements [11–13]. We used the same cut-off values of CP as these previous studies (≤30 mL for LVEDV and LVESV and ≤ 10% for LVEF). Daubert et al. [12] first reported the usefulness of CP for quality control of echocardiography. They reported that the CP for LVEDV was significantly improved after training; however, the CP for LVEF was excellent without training. These results suggest that intra-institutional variability of visual EF is very low under the supervision of an expert in each hospital, because the EF calculation is usually affected by visual EF. Thus, determination of visual EF is crucial to calculate the accurate EF. However, the authors did not report the measurement accuracy because of a lack of a reference value for LVEF. Thavendiranathan et al. [15] reported that using CMR in a training program is effective for improving inter-observer variability and accuracy of visual EF assessment. We also used CMR in our training program; however, we calculated the CP to assess the differences between 2DE and CMR measurements rather than to assess inter-observer variability so as to determine the accuracy of LV volume and LVEF calculation in each participant and at each institution. Although the Intersocietal Accreditation Commission accreditation process recommends routine assessment of echocardiographic variability and the use of a quality improvement program [23], there is no commonly accepted approach. We believe that our approach may be an effective way to achieve quality improvement both individually and inter-institutionally.

### Study limitations

This study has several limitations that should be acknowledged. First, we had no data concerning the longitudinal effect of the training program. We do not know how long the effect of training remains, and thus, it is difficult to determine when retraining is required to maintain the participants’ measurement ability at a constant level. The longitudinal effect may be influenced by the experience of each sonographer; thus, further study is required to address this point. Second, our intervention only focused on manual tracing of the LV endocardial border, which is only one aspect of quality control. Patient selection and image quality could also affect the outcome, although we used propensity score matching for the selection of the patients. Third, we used one ultrasound vendor’s machine to acquire all of the 2DE datasets. Therefore, our results cannot be generalized to other ultrasound vendors [24]. Fourth, when adopting this method into clinical practice, the reproducibility of CMR measurements is also an important consideration, since not all images are of good quality, even with CMR. Fifth, it has not been settled whether measurements of echocardiography should be similar to those of CMR. Several previous studies using echocardiographic indices showed significant prognostic value even if LV volumes and LVEF were significantly different from those of CMR measurements [25–28]. However, we believe that there should be a reference modality, such as CMR to improve the nationwide reproducibility of echocardiographic measurements [29]. The reproducibility of CMR measurements are usually better than that of 2DE [30]. Although on an echocardiogram it is easier to trace the blood-tissue boundary than to trace the interface of a compacted layer, we believe that the endocardial border visualized on a CMR is more obvious than the blood-tissue boundary on an echocardiogram, thereby making it ideal for providing objective answers to measurements. Sixth, although both CMR and echocardiography used long-axis views for measuring LV volumes, and LVEF, the number of imaging plane used for analysis was different, which made some discrepancies. Seventh, since the training was arbitrary, the lack of standardization of the practice period after the training intervention and the lack of objective evaluation were other limitations of this study. Finally, sample size was not enough to draw definite conclusions. Therefore, further studies should be required to determine the optimal number of cases and period of training.

## Conclusion

A training program for 2DE determined LV volumes and LVEF incorporating CMR as a reference improved the accuracy of measuring LV volumes and LVEF in the majority of participants. However, the degree of the learning effect differed among the hospitals, and reproducibility even became worse in two of five hospitals after the intervention. To improve accuracy and reproducibility, individualization of the training program and a periodical objective evaluation in each subject for each hospital with a standardization of the practice period is required.

## Supplementary information

**Additional file 1:** **Table S1.** Difference of echocardiographic measurements between hospitals

## Data Availability

The datasets used and analyzed during current study are available from the corresponding author on reasonable request.

## Abbreviations

2DE: Two-dimensional echocardiography
CMR: Cardiac magnetic resonance
CP: Coverage probability
CV: Coefficient of variation
LV: Left ventricular
LVEDV: Left ventricular end-diastolic volume
LVEF: Left ventricular ejection fraction
LVESV: Left ventricular end-systolic volume
SSFP: Steady-state free precision
