# Supplementary material for: Impact of a training program incorporating cardiac magnetic resonance imaging on the accuracy and reproducibility of two-dimensional echocardiographic measurements of left ventricular volumes and ejection fraction
Source: Cardiovasc Ultrasound. 2019 Oct 31;17:23. doi: 10.1186/s12947-019-0173-z (PMC6824039; doi:10.1186/s12947-019-0173-z)
Supplement: Supplementary file 1 — Additional file 1: Table S1. Difference of echocardiographic measurements between hospitals [file 12947_2019_173_MOESM1_ESM.docx]

Additional file 1: Table S1: Difference of echocardiographic measurements between hospitals

| **Protocol 1 (n=15)** | Site A | Site B | Site C | Site D |
| --- | --- | --- | --- | --- |
| **EDV** |  |  |  |  |
| Site B | 1.000 | - | - | - |
| Site C | 1.000 | 0.120 | - | - |
| Site D | <0.001† | <0.001† | <0.001† | - |
| Site E | <0.001† | <0.001† | <0.001† | 1.000 |
| **ESV** |  |  |  |  |
| Site B | 1.000 | - | - | - |
| Site C | <0.001† | 0.028† | - | - |
| Site D | <0.001† | 0.005† | 0.001† | - |
| Site E | <0.001† | 0.004† | <0.001† | 0.078 |
| **EF** |  |  |  |  |
| Site B | 0.133 | - | - | - |
| Site C | <0.001† | <0.001† | - | - |
| Site D | <0.001† | <0.001† | 0.034† | - |
| Site E | <0.001† | 0.009† | 0.090 | <0.001† |
| **Protocol 2 (n=14)** | Site A | Site B | Site C | Site D |

| **EDV** |  |  |  |  |
| --- | --- | --- | --- | --- |
| Site B | 0.867 | - | - | - |
| Site C | <0.001† | <0.001† | - | - |
| Site D | 0.073 | <0.001† | <0.001† | - |
| Site E | 1.000 | 0.002† | <0.001† | 0.011† |
| **ESV** |  |  |  |  |
| Site B | 0.467 | - | - | - |
| Site C | <0.001† | 0.029† | - | - |
| Site D | 0.010† | <0.001† | <0.001† | - |
| Site E | 1.000 | 0.032† | <0.001† | <0.001† |
| **EF** |  |  |  |  |
| Site B | 0.375 | - | - | - |
| Site C | 1.000 | 1.000 | - | - |
| Site D | <0.001† | <0.001† | <0.001† | - |
| Site E | 1.000 | 0.168 | 1.000 | <0.001† |

†: p value < 0.05

P-values were corrected by the Bonferroni method.

EDV, end-diastolic volume; ESV, end-systolic volume; EF, ejection fraction; CMR, cardiac magnetic resonance.
